# Exploring the experience of residents during the first six months of family medicine residency training

**Published:** 2017-02-24

**Authors:** Dawn Martin, Louise Nasmith, Susan Glover Takahashi, Bart J. Harvey

**Affiliations:** 1 Faculty of Medicine, University of Toronto, Ontario, Canada; 2 University of British Columbia, British Columbia, Canada; 3 Dall Lana School of Public Health, University of Toronto, Ontario, Canada

## Abstract

**Background:**

The shift from undergraduate to postgraduate education signals a new phase in a doctor’s training. This study explored the resident’s perspective of how the transition from undergraduate to postgraduate (PGME) training is experienced in a Family Medicine program as they first meet the reality of feeling and having the responsibility as a *doctor*.

**Methods:**

Qualitative methods explored resident experiences using interpretative inquiry through monthly, individual in-depth interviews with five incoming residents during the first six months of training. Focus groups were also held with residents at various stages of training to gather their reflection about their experience of the first six months. Residents were asked to describe their initial concerns, changes that occurred and the influences they attributed to those changes.

**Results:**

Residents do not begin a Family Medicine PGME program knowing what it means to be a Family Physician, but learn what it means to fulfill this role. This process involves adjusting to significant shifts in responsibility in the areas of Knowledge, Practice Management, and Relationships as they become more responsible for care outcomes.

**Conclusion:**

This study illuminated the resident perspective of how the transition is experienced. This will assist medical educators to better understand the early training experiences of residents, how these experiences contribute to consolidating their new professional identity, and how to better align teaching strategies with resident learning needs.

## Introduction

Transitions accompany and involve change. The transition from undergraduate medicine to postgraduate (residency) medical training involves moving from being in a highly dependent learner role to a role where newly graduated, but not yet independently-licensed doctors, are delegated increasing responsibility for patient care.^1^ The transition from undergraduate to postgraduate training has been described as the most stressful transition during medical training.^2^

Studies about the voiced experiences of residents as they make the transition from undergraduate to postgraduate programs have been few, and even less has been written specifically about the experiences of Family Medicine residents. Studies about Family Medicine training has focused on communication skills and used quantitative methods to evaluate different teaching and learning methods.^3^,^4^ Previous researchers have focused on the program director’s perspective of medical students’ technical preparedness^5^,^6^ or on the specific work stressors from the postgraduate trainee’s perspective.^7^,^8^

Past researchers have used surveys and questionnaires extensively to gain insight into resident-trainees’ thoughts and feelings during their postgraduate experience, but the results are often limited or speculative as there was no opportunity to probe the residents’ answers. The results of most studies, regardless of whether they are qualitative or quantitative, provide only a snapshot of what is happening at a given moment in time and do not follow residents longitudinally.

The few researchers who have taken a qualitative approach to explore the transition of medical students into postgraduate education have identified *responsibility* as a variable contributing to change,^1^,^9^–^11^ but have not explored what the change in *responsibility* means to the residents. While some researchers have used qualitative methods to explore the experiences of graduating doctors, they have asked narrowly focused questions or predominantly examined specific skills sets.^5^,^12^,^13^ Perhaps most importantly, few studies have used focus groups and individual interviews to explore, more generally, how newly graduated doctors describe their experience during the first six months of a Family Medicine training program.

More recently, there has been interest in better understanding the transition of medical students into postgraduate programs as evidenced by increased efforts that have been made to adjust the training experience of medical students to better prepare them for this shift. As well, there is growing interest in how a physician’s professional identity develops during training and with experience. The literature does suggest that the formation of a more permanent, differentiated professional identity does take place during the postgraduate training years,^14^ however there has been little exploration and examination from the perspectives of residents regarding how the experience of training contributes to this process. Variables found to influence the development of a professional identity include role transition, socialization, and identity work.^15^ The undergraduate training experience of becoming a physician is shared and generally homogenous in Canada. Deliberate differentiation occurs at the postgraduate level where the type of work and scope of practice becomes more varied. Within the practice of medicine, the training experience and scope of practice for Family Medicine is distinct from specialty training. Broadly speaking, Family Medicine training is shorter, office based, involves continuity of care with individuals/families across their life span(s), addresses health prevention and promotion, coordinates care, and is often the point of entry for patients into the healthcare system when problems are undifferentiated. Specialty training is longer, often hospital based, involves transitory patient relationships, and tends to focus on a specific patient population and disease.

Postgraduate Family Medicine residency training presents a window of opportunity to influence the continuing development of doctors in their journey to becoming independent Family Physicians. Unfortunately, little is known about the transition from medical student to resident from the postgraduate resident’s perspective. Greater understanding of this phenomenon would better enable medical educators to optimally support residents and facilitate this process.^16^,^17^ The following question guided this study, “How do residents in a Family Medicine program describe their experience during the first six months of training?”

This question was explored more in-depth through three sub-questions (See Appendix A for a further breakdown of interview questions):

- What concerns (e.g., challenges) do residents describe during the first six months of a Family Medicine training program?
- What changes do residents describe in the first six months of a Family Medicine training program?
- Who or what influenced these changes?

## Methods

If a deeper understanding of the residents’ perspectives on their training experience was going to be constructed, dialogue with the residents needed to occur over time and in a setting where they could reflect about what their lived experiences meant to them during this transition. This was a qualitative study based on the assumption that there are multiple, socially-constructed realities and that in order to make interpretations or deepen the researchers’ understanding of the residents’ experiences, access must be gained to residents’ perspectives.^18^ Thus, by asking the residents to recount and explore their thoughts and feelings about events and activities they found to be meaningful, it would be possible to construct an understanding of their experience.^19^ It was not known ahead of time what experiences would be important to residents or what stories they would voice; therefore, it was imperative to choose a method that allowed the design to vary and emerge as new information was gained and new insights formed.^20^ This study is most closely aligned with the Straussian approach to grounded theory, where an inductive-deductive process was used to access the meanings assigned by the residents to their training experience.^21^,^22^ In this approach, as data are collected, they are reviewed, compared, and coded. As repeated ideas and themes emerge, the data are recoded and labelled; the recoded data are further analyzed and grouped into concepts.^23^ The literature is used before, during, and after data collection to refine the focus and guide analysis. Grounded theory was used as a research tool to better conceptualize the social patterns and structures through the process of constant comparison.^24^

Focus groups and individual interviews were used to provide residents from a large Canadian university with opportunities to describe and reflect on their experiences during the first six months of a two-year Family Medicine residency training program. The study took place at a teaching site that provided a program where residents were based in the Family Practice Clinic for a concentrated period of time every week throughout their two years of training.^25^

Eighteen residents agreed to take part in the study – six men and twelve women who were doctors completing their two-year Family Medicine training. Six focus groups were held at the transition point from first year and second year to capture residents at three different stages of training: three focus groups with incoming residents, two focus groups at the end of first year, and one focus group at the end of their program. Focus groups lasted an average of 90 minutes. The groups were used at the beginning of the study to explore incoming residents’ experiences as they unfolded in the first few weeks of residency and to allow residents in the later stages of Family Medicine training to reflect back on their experience during the initial six months of their training. Focus groups were used to develop themes, help articulate more focused areas to be explored in the individual interviews, and later to triangulate information with other data.^26^ Five incoming Family Medicine residents took part in a series of monthly, in-depth individual interviews; the interviews were used to probe residents’ experiences in detail so that a deeper, more nuanced understanding might be developed. The focus groups and interviews were audio-taped and transcribed verbatim.

Participation in this study was voluntary and residents were reassured, both orally and through the consent form, that their decision to take part or not would in no way influence any aspect of their residency program. The ethical review protocol necessary to complete research at a medical institution (University Health Network Research Ethics Board) and as part of a university degree (OISE/UT Education Ethics Review Committee – Human Research) were submitted and approved prior to beginning the study.

## Analysis

Immediately following each interview or focus group, the data were transcribed and inductively analyzed using open coding.^27^ The findings were compared across each new case to better understand the collective experience of the residents on identified central issues, to refine lines of thinking, and to determine when saturation was reached. The process of constant comparison of data without fixed preconceptions allowed for the emergence of concepts and categories. During open coding, themes and patterns related to the concepts of *Concerns, Changes,* and *Influences* emerged and these three concepts were used to provide a general reference and direction along which to further organize the data.^28^ These concepts helped better conceptualize a multi-dimensional picture of the resident’s experience. Each concept was further analyzed looking for themes.^29^ The literature was used iteratively to locate, anchor, and triangulate the findings of the study. Multiple approaches were used (i.e., journal entries, quotes, and charts) to progressively narrow the lens moving from description to interpretation and finally to inferences. At each stage of the analysis, residents were provided with transcripts, summaries, or charts and asked to provide feedback. The data analysis was independently reviewed by three researchers at each stage of analysis, with any disagreements resolved by consensus.

## Results

By moving back and forth between the data, first looking at the concepts of Concerns, Changes, and Influences in isolation and then collectively, the subthemes of Practice Management, Knowledge, and Relationships emerged. Practice management refers to environmental activities and administrative duties such as office procedures, computers, billing, charting, and time management. Knowledge means the residents’ level of knowledge (what they knew) and how they used their knowledge in the clinical context. Relationship means the interpersonal connections to supervisors, health care professionals, peers, and patients. By deconstructing and then reconstructing the data, it was clear that the residents’ collective experience of adjusting to *responsibility* in these three areas was the core and underlying theme that anchored their collection of experiences.

### Responsibility

I feel this sense of accomplishment that I have gotten here, yet there is this enormous responsibility that goes with saying that.

**Figure 1:**
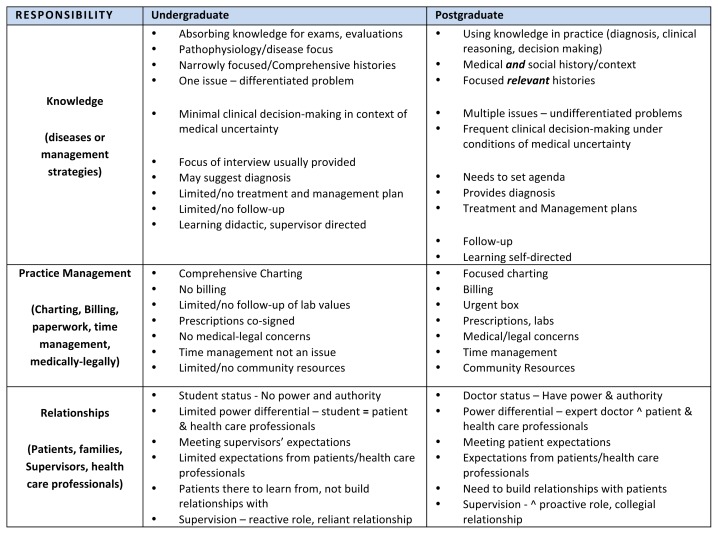
Shift in responsibility: How Family Medicine residents perceive the shift in responsibility from undergraduate to postgraduate training

The residents felt there was a huge leap in responsibility from being a medical student to being a resident. From the residents’ perspectives, they moved from the protected setting where, as a medical student, they had very limited power and authority to a setting where, as a resident, they were now responsible for the outcome of patient care. As postgraduate (residency) training commenced, the residents described being concerned with needing to manage many new responsibilities they did not have as medical students. Even though the residents may have anticipated many of their new responsibilities, the experience of both *feeling* responsible and *having* responsibility in the role of resident for the first time represented an enormous shift that caused specific concerns and changes in the areas of Practice Management, Knowledge, and Relationships (Figure 1).

### Concerns and changes

#### Practice management

Finding out if they [patients] are in the waiting room or not, and then how you are going to go out and call them and then the pieces of paper you need to get signed to get them blood work and where do you find those and there’s just so much of the system and the logistics that in the first months is the most overwhelming part.

Perhaps the most pressing concern for the residents in the first few weeks was orienting and acclimatizing to their new environment. For example, the residents had to adjust to a variety of practice management tasks (e.g., billing, booking) that would usually not have been the residents’ direct concern or responsibility when they were medical students. Adjusting to an unfamiliar work environment (e.g., locating paperwork, computer system) magnified the time pressure to manage clinical encounters. Understanding their new role, responsibilities, and associated expectations (e.g., on-call responsibilities, fully-participating member of the healthcare team) as a resident in an unfamiliar context and within an established culture (i.e., healthcare team), compounded already existing feelings of anxiety and consumed precious time and energy from other tasks (e.g., face time with patients).

Most reside*nts reported feeling far more* comfortable with “the nuts and bolts” of practice management midway into their second month. One resident recounted, “I’ve made changes in that I do try and get all of the pap [cervical cancer screening] stuff ready so I am not fumbling as they have their legs up in the stirrups.” Increased confidence in practice management issues meant resident doctors could focus more time and energy on patients and the clinical encounter. One resident described it as “… a sense of freedom … a release.”

#### Knowledge

There is an underlying concern that the patient is going to die if I don’t get the diagnosis right.

The shift into postgraduate residency training meant residents were responsible for using their hard-earned body of knowledge to diagnose and treat patients. Each and every resident reported feeling concerned about the adequacy of their level of knowledge and how to apply their knowledge in the context of the clinical encounter. Residents described feeling enormous pressure and responsibility to fulfill the patient’s expectations, which the residents initially felt was that of a medical expert. They did not feel they had the experience and medical expertise that others associated with being called a doctor. In fact, many residents described feeling as though they were “role-playing” or “masquerading” as the doctor. The fear of inadvertently “killing someone with their [inadequate] knowledge” in trying to fulfill this role dominated their thoughts and subsequent actions in their first weeks and months as a resident. Not only did they feel anxious about the adequacy of their knowledge, but they also struggled with what to ask and how to ask it.

As time passed, the residents began to change their approach to the clinical interview and to develop more effective strategies for managing the clinical encounter. For example, residents realized they needed to elicit the patients’ expectations and establish an agenda for the visit so their history taking could be more focused and discriminating. As they learned how to prioritize problems, they began asking more focused questions and became more confident in identifying the relevant “red flag” questions. When patients returned for follow up visits “unharmed,” their confidence grew as did their ability to tolerate uncertainty. At the end of six months, most residents reported feeling more consistently confident in their ability to apply their knowledge “to help, not harm.” The earlier self-imposed pressure to control the interview in order to “nail the diagnosis” was diminishing, allowing them to develop trust in taking a “wait and see” approach.

#### Relationships

As your responsibility for the patient goes up then the more actively you listen. Sometimes in clerkship I knew I had somebody covering me so I would just go in there for the experience and sit there and trail off and think of other things.

In the beginning, all residents reported feeling more concerned about harming patients with their lack of knowledge rather than establishing relationships. As a result, the focus of the relationship in the first few months became the patient’s medical diagnosis and little attention was given to the patient’s illness experience. Although they had received seminars in medical school and observed supervisors managing relational issues such as breaking bad news and inquiring about sensitive topics, being responsible themselves for holding these conversations was usually a daunting experience. “Nobody presents as the neatly labelled standardized patient you practiced on during undergraduate training.” As well, residents reported having had limited experience in medical school establishing ongoing relationships with patients*;* subsequently, knowing how to apply a patient-centered approach in a meaningful way was difficult. “You realize that the relationship wasn’t there [with patients in undergraduate training].” In the beginning, they described using a more “doctor-centered” approach to patient encounters where they needed to direct and control the interview. In contrast, towards the end of the first six months, resident doctors reported being more comfortable with sharing power and were recognizing the importance of needing to understand the patient’s perspective and background if they were to achieve optimal outcomes. Toward the end of six months, most of the resident doctors reported feeling less anxious about knowing enough to be called doctor. “Patients care more about the openness and honesty in the relationship than they do about your medical knowledge and that’s been important for me to put things in perspective.” No longer as preoccupied about their ability to treat the patient, their approach to the doctor-patient relationship shifted from being resident-centered to patient-centered.

#### Influences

I think after a bit of experience, knowing what your comfort level is, knowing how the clinic works, getting the patient back and just know what you can do … I think that’s when I began to feel, that you are their doctor.

Residents described five types of experiences they attributed to influencing the changes that occurred during the first six months of residency training: practice experience, continuity of care, time management, feedback, and role modelling.

#### Practice experience

Experience over time was the factor resident doctors most often attributed to increasing their sense of confidence in their knowledge base. “As time goes on you just start to get comfortable because you just keep seeing the same things over and over again and eventually … we have a saying - repetition teaches the donkey.” The comfort of feeling they were not going to inadvertently kill patients with their medical knowledge allowed the residents to expand their interviewing lens and to develop a more patient-centered approach. Towards the end of the first six months, most residents reported having a broader appreciation and understanding of what it meant to be a Family Physician, and resident doctors who reported feeling more confident in their knowledge base voiced this shift more clearly and earlier than others.

#### Continuity of care

Most patient care during medical school occurs in the context of single episodes, which provides limited opportunities for medical students to build relationships with patients over time and to provide follow-up care. The influence of providing care in the context of a relationship that occurred over time was pivotal to learning. “… you feel a real sense of responsibility, ownership of that relationship. These are my patients.” Not only did continuity of care allow the residents to gain confidence in their clinical decision-making and relationship-building skills, it helped them realize that not every problem had an immediate diagnosis and not every diagnosis needed an immediate solution.

#### Time management

Time was both the residents’ enemy and their friend. Knowing that the process of continuity of care allowed patients to return, the residents’ anxiety associated with needing to make immediate decisions and to manage all problems in one visit was diminished. The pressure of time constraints also influenced their approach to the clinical encounter in a positive way. “Then you realize that time is a problem and there are only so many questions that you can answer and you sort of have to make priorities.” Residents described the necessity of having an organized approach when interviewing patients, which meant learning how to prioritize problems and to be more efficient when asking questions that were more discretionary. Conversely, this same time management pressure was an ongoing source of anxiety and frustration throughout the first six months as they struggled to meet patient expectations, as well as their own.

#### Feedback

The residents described moving from a learning culture in medical school, where they were dependant on their supervisors’ feedback to gauge their progress and to act as a protective safety net. In the first few months, residents continued to rely on their supervisors’ input to gauge their performance; the supervisor’s feedback shaped the residents’ approach to practice and gave them needed confidence in their ability to doctor. As the resident doctors’ training progressed and they began developing relationships with patients, patient feedback took on an increasingly central role. “No matter how many other people tell you that you are doing a good job, sometimes that type of feedback [patient’s] cements it for you because you are actually hearing from the person that you are trying to be a doctor for.” At the end of the first six months, residents reported that patient feedback, more than supervisor feedback, helped instill a sense of confidence in their ability to use their knowledge and skills in a helpful way.

#### Role modelling

The residents recalled how, as medical students, observing both the positive and negative patient interactions of supervisors influenced who they wanted to be as doctors. However, now that they were residents, the experience of observing others interacting with patients took on increased significance because they felt responsible for the patients. “If you watch a really competent nurse who has had a lot of experience with kids giving needles all day long and maybe one kid cries, that really teaches you a lot about how to interact with children.” Learning through role modeling was anchored in personally meaningful examples, where residents had a vested interest in the outcome.

**Figure 2:**
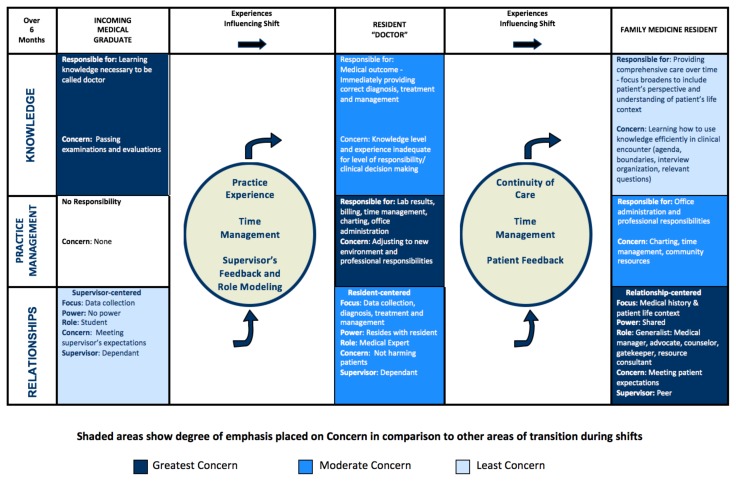
Conceptualizing the transition from undergraduate medical student to postgraduate Family Medicine Resident

Although one influence could be a more dominant or significant force for change in a given circumstance or to a specific resident, it was the combined effects of the five different influences that, over time, seemed to propel change and adjustment to practice. Through practice experience, residents were able to develop an organized approach to problems, which in turn gave them confidence in their ability to successfully care for patients. Through continuity of care and patient feedback, residents were able to see the outcomes of their treatment and management choices. A feedback loop was created that either reinforced positive results or encouraged change based on less successful outcomes. Supervisors’ and patients’ positive feedback helped residents tolerate the anxiety associated with the enormous responsibility of caring for patients and gave residents confidence in their development towards becoming Family Physicians. Watching supervisors and other health care professionals in action also helped residents learn new approaches that contributed to, or modified, their own approach. The various influences that occurred through the experience of practice changed and shaped the residents’ view of themselves and their role. The influences intersected at different points in time to shift the residents’ identities from the phases of Incoming Medical Graduate to Medical Doctor and finally to Family Medicine Resident (see Figure 2).

The following learning strategies arose from the residents’ reported experiences. None of the learning strategies suggested are new to medical educators; however, the residents were able to highlight, given their stage of development, which learning strategies were most helpful to them and when. The instructional timing and type of educational intervention is important^30^ and has received little attention from researchers.^31^ The residents’ viewpoints on their experiences help provide useful information to better target educational interventions aligned with their learning needs during the first six months of postgraduate residency training (See Table 1).

## Discussion

This study found residents do not begin a Family Medicine postgraduate residency training program knowing what it means to be a Family Physician, but they must learn what it means and how to fulfill this role. From the residents’ perspective, this process begins with feeling more comfortable and confident in the role of doctor. Identifying with, and owning the role of doctor, involves adjusting to significant shifts in responsibility in the areas of Knowledge, Practice Management, and Relationships, which occurs when they make the transition from being an undergraduate medical student to a graduate-resident. For the first time, graduate residents now *have* and *feel* responsibility for the outcome of patient care. As the residents began postgraduate residency training they were eager to accept the responsibility of being the doctor, but were uncertain they had the necessary medical knowledge, experience, and expertise for someone calling themselves “doctor.” As the residents adjusted to their new responsibilities, they gained confidence in their new role as doctor, which subsequently led to a more comprehensive understanding of what it meant to become a Family Physician.

Many of the results of this study support the findings of other studies and add another layer to the understanding of this pivotal transition toward independent practice. Newly graduated doctors’ perceptions of their preparedness for postgraduate residency training is influenced by their experiences during medical school.^33^ The transition between undergraduate to postgraduate training represents a huge leap in responsibility.^34^ There is clearly an evolving, reconfiguring of professional identity occurring as newly graduated doctors adjust to both having and feeling responsible for the outcome of patient care, which, from their perspective, did not exist during medical school.^35^,^36^

Adapting to a new level of responsibility is hard and it takes time to adjust to the new role.^37^,^38^ This study’s findings mirror those of other studies in that transitions often raise feelings of anxiety and insecurity from not fully knowing how to adapt to a new professional role and work environment.^39^–^41^ However, in this study, residents appeared to be as concerned about their level of knowledge being adequate as they were about coping with their new roles and tasks. One explanation for this finding is that the study focused on the experiences occurring in a Family Medicine context. In Family Medicine, patients often present with undifferentiated symptoms and complex/unfocused problems, unlike other specialties where the diagnosis is often predetermined or focused. Becoming a physician involves changes in professional identity which occur over time.^40^ One of the changes involves learning how to manage medical uncertainty. Learning how to take “a wait and see” approach is a key part of becoming a Family Physician. Perhaps new Family Medicine residents feel earlier, and more acutely, the pressure to manage the medical uncertainty that comes with being a physician; therefore, it was more a focus of their reflections. Another reason might be that Family Medicine residents immediately inherit a patient roster from a graduating Family Medicine resident where they are immediately responsible for providing continuity of care, unlike many other specialties where patient contact can be more time limited and transitory. Family Medicine residents may feel a different type of responsibility because they have an established practice earlier. As well, because they have longitudinal relationships, they may feel more responsible for meeting their patients’ expectations. Additionally, it may be relevant that the context for this study was a Canadian University where the Family Medicine program is two years in duration.^42^ Family Medicine residents may feel additional time pressure related stress to master their profession, compared to specialty residents who have a longer duration to grow into their professional identity. The different roles, type of work, patient exposure, and length of program that distinguishes Family Medicine from the other specialties might account for how the workplace is experienced and accounted for. 43,44

Moreover, what do these findings mean in light of the trainee who does not feel comfortable and confident in the role of doctor after six months into residency training? The results of this study suggest the trainees’ broader identification with the role of Family Medicine resident is not halted, but slowed. As well, not identifying with the role of doctor at a particular time juncture may suggest that the resident struggles with feeling confident in their knowledge base, and may need additional learning support.

### Limitations and next steps

As mentioned, residency training in Canada to become a Family Physician is a two-year process. This study explored the first six months and not the subsequent eighteen months nor the experiences of the residents as they entered independent practice after completing their postgraduate residency training. This study underscores that residents at different junctures in their training and practice may have different experiences to reflect on and therefore, different perspectives to contribute. Although all residents interviewed through focus groups were asked to reflect on the first six months, those residents in their later stages of their Family Medicine training might have recall bias toward their earlier experiences.

This study focused on the development of postgraduate trainees in a Family Medicine program. The training to become a general surgeon, pathologist, paediatrician, or internist involves different types of work and work experience. Further research is needed to determine how the experience in other medical specialties may be different or similar from that of Family Medicine. The experience of becoming a competent physician in any discipline or specialty is complicated, but the more medical educators listen to resident voices, the more they will understand about this transition and in doing so, be better able to facilitate a smoother journey.

Residents often assume that they are alone in experiencing anxiety and self-doubt, and medical educators sometimes struggle with knowing how best to support their transition. The findings of this study provide both medical educators and residents setting out in a Family Medicine postgraduate residency training program with markers for better locating and understanding of the experience of training during the first six months.

## Figures and Tables

**Figure 3:**
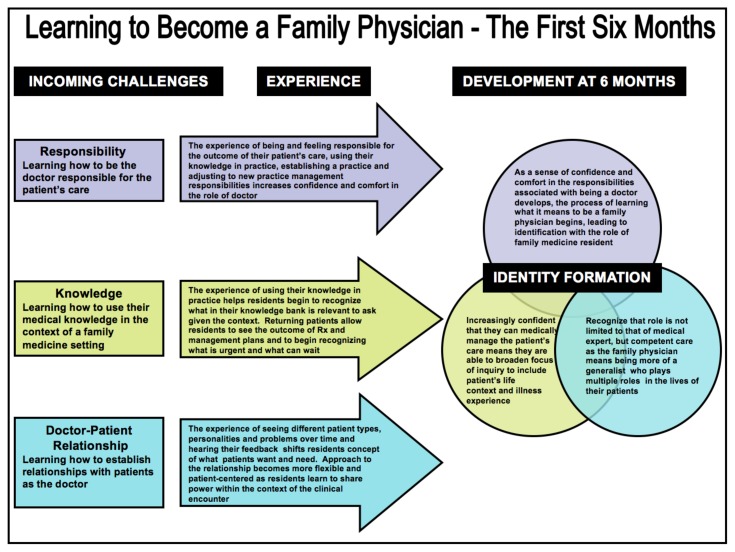
“Learning to Become a Family Physician – The First Six Months” is used to not only synthesize findings, but to lift them to a more inductive level providing a deeper understanding of how the experience of the first six months of a postgraduate Family Medicine program shapes the resident’s professional identity

**Table 1:** Alignment of learning strategies during the first six months of Family Medicine training

Struggle with …	Implication	Learning Strategy	Benefit

**Comfort and confidence in role of doctor**	Transitory relationships where they don’t have or feel continuing responsibility for patients Masquerading as doctor because feel knowledge base is not reflective of a ‘real’ doctor. Preoccupied with harming patients rather than building relationships	Continuity of Care Experiences	Returning patients increase opportunities to observe the link between patient perspective and life context to optimize outcomes, as well as, to hear patient feedback.
		Urgent care Experiences	Urgent care consolidates knowledge through reinforcement of ‘Red Flag” questions

**Time Management**	Anxiety provoking and frustrating, contributes to feelings of inadequacy	Practical strategies shared by staff e.g. get equipment ready ahead of time, agenda setting	Shapes approach to practice and increases feeling of being in control.

**Practice Management**	Stress as the newcomer, compounds feelings of anxiety and interferes with time management. Takes away from patient focus.	Introduce the healthcare team. Make explicit theirs and others roles and responsibilities. Establish work expectations. Orient to the physical layout. Review setting protocols e.g. scheduling, EMR	Feel more grounded in role of doctor. Able to focus more time and energy on patient care.

**Gauging their performance**	Assumption that residents know/recognize the behaviour, attitudes, rationale, or thinking intended by staff and the right learning message will be noted through casual observation.	Provide verbal reassurance and regular feedback about positives, as well as, areas for improvement. Share tacit thinking e.g. treatment choices, approach. Actively seek and deliberately create opportunities to role model the behaviour and attitudes you want to impart e.g., boundary setting, use of resources. Explicitly label your choices and behaviors	New residents are highly motivated to learn, anxious about their abilities, and receptive to guidance. Reduces resident’s anxiety and uncertainty.

**Setting agendas, collecting relevant history, and presenting organized treatment and management plans.**	Affected other areas of their practice, such as clinical decision-making and gaining confidence in using a more interactive approach.	Conceptual Interview framework Seminars in focused topic/skill areas e.g., boundary setting, setting agendas	Providing undergraduate and postgraduate medical trainees with a conceptual framework that reflects how practising physicians conduct and structure organized, patient-centered interviews from beginning to end such as Martin’s Map^32^ might assist medical trainees, regardless of training level, how to conceptualize how to use what they know.

## Appendix A

### Examples of interview questions

**Table:**

Describe a patient encounter.
Describe a challenging situation.
How has the past month been going?
Do any moments stand out for you in the past month?
Describe your approach to the clinical encounter.
How do you think patients see you?
What has it been like caring for your own patients?
What are some of the good things about seeing returning patients? Not so good things?
Describe your role in the doctor-patient relationship.
What has been your biggest surprise? Frustration? Struggle?
How have you changed in the past few months?
Has your role changed?
Describe your relationship with your supervisor.
